# Electrothermal Failure Physics of GaN Schottky Diodes Under High-Temperature Forward Biasing

**DOI:** 10.3390/mi16030242

**Published:** 2025-02-20

**Authors:** Nahid Sultan Al-Mamun, Yuxin Du, Jianan Song, Rongming Chu, Aman Haque

**Affiliations:** 1 Department of Mechanical Engineering, Penn State University, University Park, PA 16802, USA; nma5621@psu.edu; 2 Department of Electrical Engineering, Penn State University, University Park, PA 16802, USA; yqd5203@psu.edu (Y.D.); jus824@psu.edu (J.S.)

**Keywords:** GaN Schottky diode, in situ TEM, high-temperature operation of GaN, electrothermal degradation of GaN, reliability of GaN

## Abstract

The reliability of GaN-based devices operating under high temperatures is crucial for their application in extreme environments. To identify the fundamental mechanisms behind high-temperature degradation, we investigated GaN-on-sapphire Schottky barrier diodes (SBDs) under simultaneous heating and electrical biasing. We observed the degradation mechanisms in situ inside a transmission electron microscope (TEM) using a custom-fabricated chip for simultaneous thermal and electrical control. The pristine device exhibited a high density of extended defects, primarily due to lattice mismatch and thermal expansion differences between the GaN and sapphire. TEM and STEM imaging, coupled with energy-dispersive X-ray spectroscopy (EDS), revealed the progressive degradation of the diode with increasing bias and temperature. At higher bias levels (4–5 V) and elevated temperatures (300–455 °C), the interdiffusion and alloying of the Au/Pd Schottky metal stack with GaN, along with defect generation near the interface, resulted in Schottky contact failure and catastrophic device degradation. A geometric phase analysis further identified strain localization and lattice distortions induced by thermal and electrical stresses, which facilitated diffusion pathways for rapid metal atom migration. These findings highlight that defect-mediated electrothermal degradation and interfacial chemical reactions are critical elements in the high-temperature failure physics of GaN Schottky diodes.

## 1. Introduction

Wide-bandgap gallium nitride (GaN)-based devices have emerged as a transformative technology in the semiconductor industry due to their exceptional transport properties and high critical electric field. These characteristics enable their superior performance in high-power, high-frequency, and high-temperature applications [1,2,3,4]. GaN offers significant advantages over traditional silicon-based devices and other wide- and ultrawide-bandgap materials, making it ideal for extreme environments such as nuclear reactors, automotive systems, aerospace applications, spacecraft, and satellites [3,5,6,7]. GaN-based technologies have been successfully integrated into various power electronic devices for commercial applications, including metal–semiconductor field-effect transistors (MESFETs), high-electron-mobility transistors (HEMTs), and diodes [8,9,10,11]. Among these, GaN Schottky diodes are particularly attractive for switching circuits and high-power rectifier applications because of their low forward voltage drop and high breakdown voltage [12,13].

Despite their promising potential, GaN devices have yet to achieve long-term reliable operation, primarily due to the high power density and elevated junction temperatures in the active regions of the devices [14,15,16]. The challenge of producing large-area, low-cost single-crystal GaN substrates necessitates the heteroepitaxial growth of GaN on foreign substrates such as silicon (Si), silicon carbide (SiC), and sapphire (Al_2_O_3_) [17,18], despite the potential better reliability of devices on free-standing GaN substrates [19]. However, this heteroepitaxial growth introduces a high density of defects, including threading dislocations, stacking faults, and point-defect complexes, which can serve as leakage pathways or nucleation sites for degradation, ultimately compromising device performance [20,21,22,23]. These defects often originate at the GaN/substrate (Si/SiC/Al_2_O_3_) interface due to the substantial lattice mismatch and differences in thermal expansion coefficients. This mismatch generates misfit dislocations and residual strain during epitaxial growth and subsequent cooling [24,25]. In high forward or reverse bias applications, threading dislocations can promote localized degradation and device failure [26,27]. A high-temperature environment exacerbates the degradation process by introducing significant thermoelectric stress, added to that from Joule heating [28,29]. GaN-on-sapphire devices can experience additional thermal stress due to the relatively low thermal conductivity of the sapphire [30,31].

A significant challenge in GaN Schottky diodes lies in maintaining the stability of the metal–semiconductor interface and ensuring the reliability of the Schottky contact stack, typically composed of Ni/Au-, Pt/Au-, or Pd/Au-based systems [29,32,33,34]. At elevated temperatures and high current densities, interdiffusion, alloy formation, and electromigration can become prominent, compromising the integrity of the contact [29,35,36]. These interfacial reactions can lead to catastrophic device failure if they extend deep into the GaN lattice, resulting in the formation of gallides or other stable intermetallic compounds [36,37,38,39,40,41].

To develop effective mitigation strategies and enhance device reliability, a fundamental understanding of electrothermal degradation mechanisms is crucial. Conventional ex situ analysis techniques—such as scanning or transmission electron microscopy, secondary ion mass spectrometry, Raman spectroscopy, cathodoluminescence, and X-ray diffraction—only provide post-failure snapshots. This limitation makes it challenging to capture transient processes or determine the precise sequence of microstructural transformations [42,43,44,45,46,47]. In contrast, in situ transmission electron microscopy (TEM) offers a unique capability for direct, real-time observations of structural, morphological, and compositional changes, as well as local strain fields, within the material. This approach enables the study of dynamic processes at the nanoscale, providing valuable insights into the mechanisms driving the degradation [37,38,48,49,50,51,52].

In this work, we present an in situ TEM study of a GaN Schottky diode subjected to simultaneous heating and electrical bias to investigate the mechanisms driving device degradation. A custom-designed microelectromechanical system (MEMS) chip, capable of applying both temperature and voltage simultaneously, was employed to capture dynamic microstructural changes in the GaN diode across varying bias levels until catastrophic degradation. High-resolution TEM (HRTEM) imaging, scanning TEM (STEM), and energy-dispersive spectroscopy (EDS) were utilized to identify the formation of new defects, the interdiffusion of Schottky metallization into the GaN layer, and microstructural breakdown phenomena. These findings were further correlated with geometric phase analysis (GPA) strain maps, offering detailed insights into the emergence and evolution of localized strain fields and defect networks under escalating electrothermal stress.

## 2. Materials and Methods

GaN Schottky diodes were fabricated on a 4-inch GaN-on-sapphire epitaxial wafer grown using metalorganic chemical vapor deposition (MOCVD) by a commercial vendor. The epitaxial structure, from bottom to top, includes a GaN buffer layer, a 300 nm intrinsic GaN (i-GaN) channel, a 100 nm Si-doped n-GaN channel with a doping concentration of 5 × 10^17^ cm^−3^, a 40 nm unintentionally doped i-GaN spacer, a 100 nm Mg-doped p-GaN layer with a doping concentration of 1 × 10^19^ cm^−3^, and a 10 nm p+-GaN layer with an Mg doping concentration of 1 × 10^20^ cm^−3^. Magnesium activation was achieved through annealing in N_2_ at 800 °C for 10 min.

**Figure 1:**
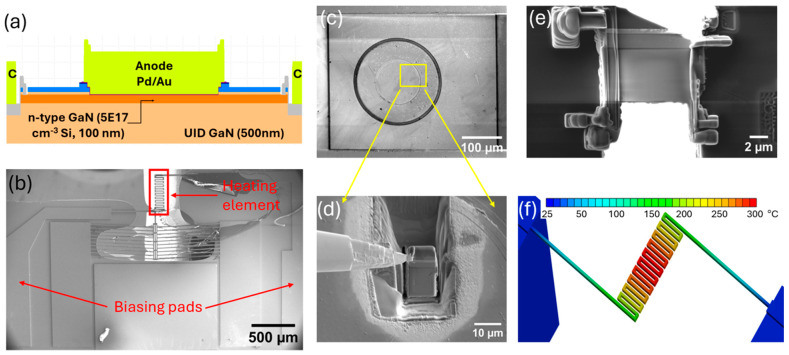
(**a**) Schematic cross-section of the GaN SBD; (**b**) SEM top view of the MEMS chip; (**c**–**e**) FIB lamella preparation and transfer of the diode lamella into the MEMS microheater element; (**f**) Multi-physics simulation result of thermal field of the MEMS microheater element at 4 V.

The device fabrication process began with 400 nm isolation etching, followed by aluminum implantation. The p+-GaN and p-GaN layer was partially etched 60 nm for E-field handling. Additional 90 nm trench etching stopped at the top of the n-GaN layer to form the n-ohmic contact and the Schottky barrier diode. The p-ohmic contact was created by evaporating Pd/Au (50/50 Å) onto the p-GaN layer. Ti/Al (200/1200 Å) was deposited in the trench to create a combination of planar and sidewall n-ohmic contacts, followed by 1 min N_2_ rapid thermal annealing at 550 °C. Finally, Pd/Au metal (200/5000 Å) was used for the anode and cathode contacts. A schematic cross-section of the diode is shown in Figure 1a.

A custom-made MEMS chip was utilized for in situ TEM heating and biasing experiments. The MEMS chip was fabricated on a silicon-on-insulator (SOI) wafer with a 20 μm device layer, a 2 μm buried oxide (BOX) layer, and a 400 μm handle layer using conventional nanofabrication techniques, including photolithography, device layer reactive-ion etching (RIE), handle layer deep reactive-ion etching (DRIE), and wet etching of BOX layer using buffer oxide etching (BOE) chemistry. The chip design incorporated integrated microheater elements and electrical contacts to enable simultaneous heating and electrical biasing during TEM analysis. An SEM top view of the MEMS chip is shown in Figure 1b.

A ~3 μm thick lamella was extracted from the bulk diode using FEI Scios-2 DualBeam focused ion beam (FIB) system equipped with gallium ion (Ga+) source. The diode lamella was transferred into the MEMS chip using the FIB in situ manipulator needle (OmniProbe). The lamella was carefully welded into microheater elements using a platinum-based gas injection system (GIS) to ensure mechanical stability and electrical connectivity. The lamella’s extraction and transfer to the MEMS chips are shown in Figure 1c–e. A triangular shape was cut on the upper section of the lamella to prevent current flow through the protective carbon layer. The diode lamella was then thinned down to ~120 nm through a series of FIB milling steps at progressively lower beam currents and voltages ranging from 1 nA to 78pA and 30 kV to 5 kV, respectively. To minimize the beam-induced damage, final polishing was carried out at 2 kV. The MEMS chip containing the diode lamella was mounted onto a Protochips in situ biasing and heating TEM holder. The in situ experiment was conducted inside a field emission 200 kV FEI Talos F200X TEM, equipped with EDS and STEM detectors. The diode lamella was biased through the MEMS chip at 1, 2, 3, 4, and 5 V for 60 s. A relaxation time of 30 min was allowed in between each subsequent biasing level to prevent any residual heating effect of the previous biasing condition. The microheater element of the MEMS chip simultaneously generates a temperature of 45, 85, 185, 300, and 455 °C at 1, 2, 3, 4, and 5 V of biasing. Ansys multi-physics simulation was conducted simulating the actual vacuum environment inside the TEM to estimate the temperature rise on the microheater element of the MEMS chip at different biasing levels, as shown for 4 V in Figure 1f.

## 3. Results and Discussion

**Figure 2:**
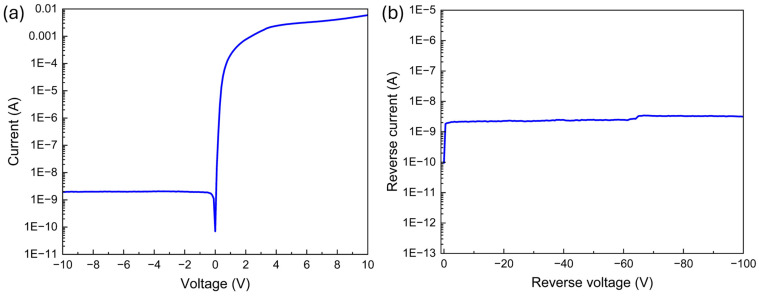
(**a**) Forward I–V and (**b**) high-voltage reverse I–V curves of the fabricated SBD.

The electrical characteristics (i.e., forward I–V and high-voltage reverse I–V curves) of the fabricated GaN Schottky diodes are shown in Figure 2. The fabricated diodes show good rectification behavior and stability up to −100 V of reverse bias at room temperature.

**Figure 3:**
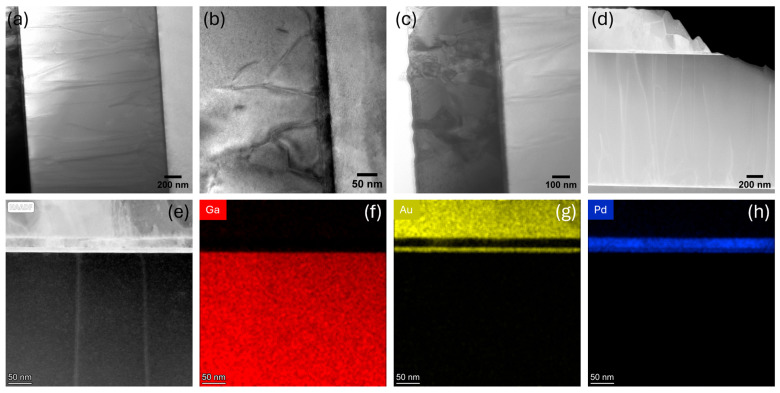
(**a**) Low-magnification TEM bright-field image of pristine GaN diode; (**b**) dislocations near the GaN/sapphire interface; (**c**) extension of dislocations towards anode; (**d**) STEM image of the pristine diode; (**e**) HAADF STEM image near the anode; EDS maps of (**f**) Ga, (**g**) Au, and (**h**) Pd.

The bright-field TEM cross-sections of the pristine diode are shown in Figure 3a–c. The pristine device contains a lot of extended defects in the GaN layer, which are visible in the low-magnification TEM image (Figure 3a). These defects are nucleated at the GaN/sapphire interface, as shown in Figure 3b, and many of these defects are extended to the anode of the diode, as shown in Figure 3c. The significant lattice mismatch (approximately 16%) and the difference in thermal expansion coefficients between the GaN and sapphire result in the formation of a high density of extended defects in the GaN layer. The lattice mismatch induces strain at the GaN/sapphire interface during the early stages of GaN growth. This strain is relieved through the formation of misfit dislocations, which can extend into the GaN layer as threading dislocations [20]. Furthermore, the thermal expansion coefficient mismatch between the GaN and sapphire generates thermal stress during the post-growth cooling process, further promoting the formation of extended defects, including threading dislocations and stacking faults [53,54]. The bright-field STEM image (Figure 3d) further confirms the presence of such defects in pristine condition. These extended defects in the GaN layers negatively impact the performance of the Schottky diode by increasing the leakage current, as threading dislocations act as current paths under reverse bias and those with a screw dislocation component contribute to leakage under forward bias [55,56]. They also shift the turn-on voltage by changing the Schottky barrier height [27,57]. Additionally, these defects reduce the breakdown voltage by causing localized electric field enhancements, leading to the premature breakdown of the device [58,59]. The EDS maps of the anode Schottky region along with the high-angle annular dark field (HAADF) STEM image are shown in Figure 3e–h. The pristine device exhibits a well-defined sharp interface between the GaN and Au layers, as well as a distinct Au/Pd/Au stacking structure for the anode metals.

**Figure 4:**
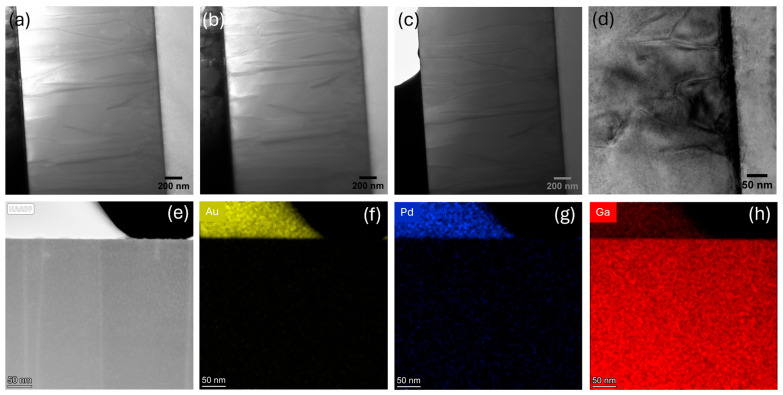
Low-magnification TEM cross-section after biasing at (**a**) 2 V and 85 °C, (**b**) 3 V and 185 °C, and (**c**) 4 V and 300 °C; (**d**) new defect generation near substrate; (**e**) HAADF image near the damaged Schottky contact; (**f**) Au, (**g**) Pd, and (**h**) Ga EDS maps at 4 V and 300 °C.

The GaN diode lamella was biased through the MEMS chip at different biasing voltages with simultaneous heating. The low-magnification images do not show any noticeable change in the microstructure of the diode after biasing at 1, 2, and 3 V, with simultaneous heating at 45 °C, 85 °C, and 185 °C, respectively (Figure 4a,b). The partial failure of the anode Schottky contact at the edge is observed at 4 V and 300 °C, as shown in Figure 4c. The electric field in a Schottky contact is stronger at the edges due to field crowding and edge effects [60]. The electric field lines concentrate near the contact boundary because of the geometry, leading to a non-uniform distribution. Additionally, surface states at the metal–semiconductor interface can trap charges, further distorting the electric field distribution and increasing the field strength near the edges [61]. Clusters of new defects covering a wide region are also observed near the GaN/sapphire interface, as shown in Figure 4d. The defect generation at the GaN/sapphire interface during high-temperature biasing is primarily due to the thermal expansion coefficient mismatch between the GaN and sapphire. In addition, the poor thermal conductivity of sapphire (~35 W/m·K) inhibits proper heat dissipation, leading to localized heating and additional thermal stress. The darker contrast in Figure 4d possibly indicates the high-temperature biasing-induced non-uniform strain distribution in the GaN layer, which results from the accumulation of defect clusters.

The HAADF STEM image reveals the generation of an additional threading dislocation in the GaN layer under the damaged Schottky contact, as shown in Figure 4e. Instead of the distinct layers seen in the HAADF STEM image of the pristine device near the anode (Figure 3e), a single, coalesced Au/Pd layer is formed after biasing at 4 V and 300 °C. High-temperature biasing can significantly reduce the diffusion barrier of the thermally activated mobile metal atoms to promote intermixing and defect-enhanced diffusion. The grain boundaries and dislocations, which are prevalent on the Au and Pd layers of the pristine device (Figure 3c,e), act as the dominant pathways for accelerated diffusion at elevated temperatures [62]. Additionally, close lattice constants with similar crystal structures of Au and Pd (~5% lattice mismatch) favor the formation of substitutional solid solutions and alloying [63]. A noticeable interdiffusion of a Au/Pd bilayer system has been reported at a temperature higher than 200 °C [62]. As the temperature increases above this threshold, the diffusion coefficients rise sharply and alloying becomes increasingly rapid with time. Moreover, higher current densities enhance Au/Pd intermixing through combined electromigration-driven mass transport and elevated temperatures caused by Joule heating, ultimately accelerating the breakdown of the initially well-defined interface of the Au/Pd stack [64]. Here, the complete intermixing and alloying of Au/Pd are confirmed by the EDS maps in Figure 4f,g at 300 °C at 4 V. The EDS map of Ga also demonstrates a small amount of Ga out-diffusion into the Schottky metal stack (Figure 4h). The out-diffusion of Ga from GaN into Au/Pd Schottky metallization at an elevated temperature primarily occurs due to the thermodynamically stable intermetallic compounds’ formation of Ga with Au and Pd at the GaN and alloyed Au/Pd interface [41,65,66]. The formation of stable gallides creates a chemical potential gradient that pulls Ga out of the semiconductor and into the contact layers.

**Figure 5:**
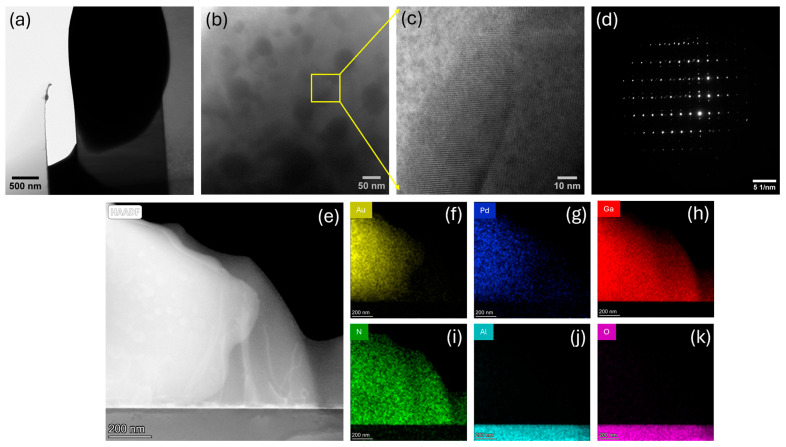
(**a**) Failure of Schottky contact at 5 V and 455 °C; (**b**,**c**) Au/Pd nanocrystallites in the GaN layer; (**d**) SAED pattern of GaN layer after metal diffusion; (**e**) HAADF STEM image shows widespread diffusion of metal into GaN; EDS maps of (**f**) Au, (**g**) Pd, (**h**) Ga, (**i**) N, (**j**) Al, and (**k**) O.

As the biasing voltage and temperature are increased further to 5 V and 455 °C, respectively, severe degradation of the diode is observed with the catastrophic failure of the Schottky contact (Figure 5a). At an elevated temperature, the previously alloyed Au/Pd readily diffuses into the GaN due to thermally activated interfacial reactions and forms stable Au-Ga and Pd-Ga alloys or metal–nitride compounds. Many of those diffused metals are dispersed randomly into the GaN matrix as Au/Pd nanocrystallites, as observed in Figure 5b,c. The nonuniform diffusion of Au and Pd atoms and their limited solubility in the GaN matrix cause locally super-saturated regions at certain spots, specifically at the vicinity of defects, forming stable precipitates of Au/Pd. The selected area electron diffraction (SAED) pattern of the GaN layer (Figure 5d) reveals the presence of extra diffraction spots in the crystal along with the bright spots of the GaN crystal. However, due to the close lattice parameters of Au and Pd, the mutual alloying of these elements with GaN, and the intermixing of different zone axes, it was not possible to accurately index the diffraction spots of the SAED pattern.

The HAADF STEM image (Figure 5e) along with the EDS maps (Figure 5f–k) further confirm the widespread diffusion and intermixing/alloying of Au and Pd in the GaN layer. At an elevated temperature, the GaN surface can partially decompose or lose N, allowing Ga to migrate out of the GaN lattice and form Ga and N vacancies. Although the partial thermal decomposition of GaN in vacuum has been reported at a temperature above 700 °C [67], the effective decomposition temperature at the GaN/metal interface can be lower depending on the specifics of the metal contact, interface quality, and defect density of GaN [36,41,68,69]. The formation of gallides, i.e., Au-Ga and Pd-Ga intermetallic phases, were reported at a temperature above 500 °C and 600 °C, respectively [36,41,69]. Although the widespread diffusion of Au/Pd into the Ga layer was observed in the presence of an external heating of 455 °C, the actual temperature of the GaN diode on the sapphire substrate could be significantly higher due to the high current density inducing the high joule heating of the thin diode lamella at 5 V of biasing. The metal diffusion process is often mediated by the presence of vacancies, interstitials, dislocations, and defect complexes at elevated temperatures. The GaN layer in pristine condition already contains a large number of defects, as shown in Figure 3. The high joule heating accompanied by the external temperature can significantly degrade the crystal quality of the GaN, further generating additional defects by localized heating due to the poor thermal conductivity of sapphire and promoting the swift diffusion of the metal atoms.

**Figure 6:**
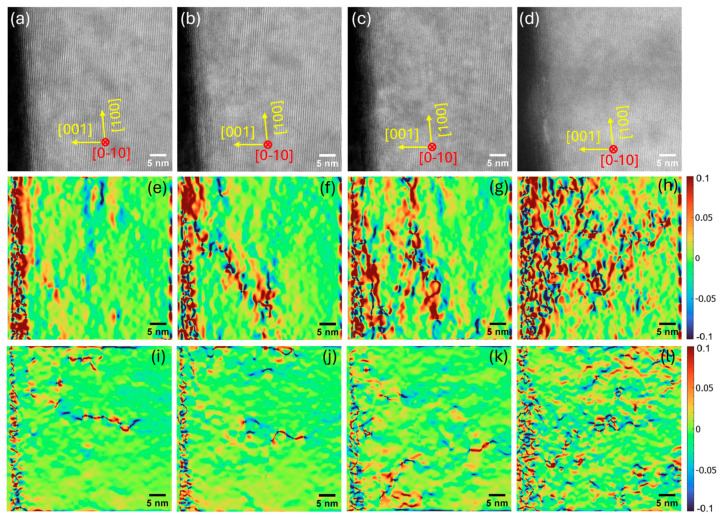
HRTEM images for (**a**) pristine, (**b**) 2 V, 85 °C, (**c**) 3 V, 185 °C, and (**d**) 4 V, 300 °C. GPA strain maps: in-plane strain maps (ε_xx_) for (**e**) pristine, (**f**) 2 V, 85 °C, (**g**) 3 V, 185 °C, and (**h**) 4 V, 300 °C. Out-of-plane strain maps (ε_yy_) for (**i**) pristine, (**j**) 2 V, 85 °C, (**k**) 3 V, 185 °C, and (**l**) 4 V, 300 °C.

To further analyze the degradation of the diode through defect generation, HRTEM images were obtained near the anode and are shown in Figure 6a–d for different biasing conditions. The HRTEM image of the pristine device (Figure 6a) reveals well-resolved atomic fringes with relatively few extended defects and a sharp interface. The HRTEM images after biasing at 2 V and 85 °C and at 3 V and 185 °C (Figure 6b and Figure 6c, respectively) show the apparently uniform lattice fringes. A slight change in contrast appears near the GaN/Schottky interface after biasing at 3V and 185 °C, suggesting the onset of point defect formation or small dislocations. A substantial defect contrast emerges throughout the GaN layer and interface region after biasing at 4 V and 300 °C (Figure 6d), manifesting as darker/brighter bands or blurry patches consistent with the aggregation of point defects, dislocations, and/or amorphized regions.

A geometric phase analysis (GPA) was performed on the HRTEM images to pinpoint the localization of defects and estimate the strain field. Defect cores, threading dislocations, and interfacial misfit commonly induce pronounced local lattice distortions. The GPA highlights these distortions quantitatively by computing the local phase or small lattice displacement and produces spatially resolved strain maps that facilitate the visualization of localized strain fields associated with each defect [70,71], even if the defects are not always as immediately obvious as in the HRTEM images. The in-plane (ε_xx_) and out-of-plane (ε_yy_) strain maps of the HRTEM images are shown in Figure 6e–h and Figure 6i–l, respectively. The strain maps of the pristine device (Figure 6e,i) are fairly uniform, indicating minimal lattice deformation. The small regions with red and blue patches are the growth-induced residual strain. Although the GaN layer of the pristine device contains several long-range dislocations extended to the anode of the diode, we purposely avoided such areas in the HRTEM image to exclude the effect of pre-existing defects and only to capture the generation of electrothermal bias-induced defects at an elevated temperature. The GPA strain maps at 2 V and 85 °C (Figure 6f,j) show some localized red/blue regions, especially near the interface, which could be due to the accumulation of pre-existing defects under the high current density of the thin diode lamella. More pronounced dislocation networks are visible at 3 V and 185 °C, indicating the possible formation of new defects. Interface roughening or a minor reaction at the GaN/metal boundary is also visible. The strain maps at 4 V and 300 °C (Figure 6h,l) show more continuous, interconnected regions of higher lattice strain around the defect clusters. The higher defect density at the interface region is the result of high-temperature bias-induced electrothermal strain. The lattice mismatch and thermal expansion coefficient mismatch at the GaN/Schottky metal interface promote extended defects at an elevated temperature, producing strong, overlapping strain fields. The d-spacing values of the GaN lattice planes for different biasing conditions, reported in Table 1, demonstrate a decreasing trend with the increase in biasing voltage and temperature, suggesting an overall increase in compressive strain. A greater compressive lattice strain indicates an increase in vacancy concentrations. Greater vacancies along with the dislocations at the interface act as the diffusion paths for the Au and Pd to diffuse into the GaN layer under high-temperature operation. The d-spacing values at 4 V and 300 °C increase slightly, which could be due to the presence of diffused Au/Pd interstitial atoms in the GaN lattice.

## 4. Conclusions

The in situ TEM study conducted in this research highlights the significant impact of combined thermal and electrical stress on GaN-on-sapphire Schottky barrier diodes. Lattice mismatch and differences in thermal expansion coefficients at the GaN/sapphire interface resulted in the formation of extended defects under pristine conditions, which were further exacerbated during biasing and heating.

No substantial microstructural changes were observed up to 3 V and 185 °C, apart from the accumulation of minor defects near the GaN/Schottky interface. However, at 4 V and 300 °C, a partial Schottky contact failure occurred at the diode edge, driven by field crowding and defect-mediated metal interdiffusion. The alloying of the Au/Pd Schottky metals was also observed at this stage. At 5 V and 455 °C, the widespread diffusion of Au and Pd into the GaN lattice led to catastrophic diode degradation. Elevated local temperatures, driven by Joule heating from high current densities and external heating, significantly enhanced the diffusion of Au and Pd into the GaN layer. The formation of stable gallides and Au–Pd alloys further compounded the severe microstructural degradation.

High-resolution TEM and the GPA strain analysis revealed the emergence of a network of newly formed defects, including vacancies and dislocations, which accelerated the degradation by providing fast diffusion pathways for metal atoms. While the simultaneous application of heating and biasing via the MEMS chip made it challenging to isolate the individual contributions of Joule heating and external heating to the degradation, the results underscore the critical role of defect-mediated processes and interfacial chemical reactions in the failure of GaN Schottky diodes. These findings emphasize the need for optimized device architectures and improved metallization schemes to enhance the thermal and electrical reliability of GaN-based devices.

## Figures and Tables

**Table 1:** The d-spacing values of GaN lattice planes for different biasing conditions in [0–10] zone axis.

	d-Spacing (nm)				
Lattice Planes	Pristine	1 V, 45 °C	2 V, 85 °C	3 V, 185 °C	4 V, 300 °C
[100]	0.2972	0.2971	0.2960	0.2920	0.2956
[101]	0.2624	0.2623	0.2597	0.2577	0.2606
[001]	0.5599	0.5592	0.5522	0.5517	0.5566
[−101]	0.2626	0.2622	0.2621	0.2586	0.2615

## Data Availability

The raw data supporting the conclusions of this article will be made available by the authors on request.
